# Thermodynamic Signatures of Blood Plasma Proteome in Neurodegenerative Pathologies

**DOI:** 10.3390/ijms24010789

**Published:** 2023-01-02

**Authors:** Avgustina Danailova, Svetla Todinova, Lidia Gartcheva, Desislava Bogdanova, Elena Zlatareva, Nikolay Kalaydzhiev, Ivan Milanov, Sashka Krumova, Stefka G. Taneva

**Affiliations:** 1 Institute of Biophysics and Biomedical Engineering, Bulgarian Academy of Sciences, “Acad. G. Bonchev” Str. 21, 1113 Sofia, Bulgaria; 2 National Specialized Hospital for Active Treatment of Haematological Diseases, “Plovdivsko pole” Str. 6, 1756 Sofia, Bulgaria; 3 University Multiprofile Hospital for Active Treatment in Neurology and Psychiatry “St. Naum”, “Louben Roussev” Str. 1, 1113 Sofia, Bulgaria

**Keywords:** blood plasma biomarkers, Parkinson’s disease, amyotrophic lateral sclerosis, differential scanning calorimetry, thermal transitions, transition temperature, excess heat capacity, globulin fractions

## Abstract

Discovery of diagnostic biomarkers for age-related neurodegenerative pathologies (NDDs) is essential for accurate diagnosis, following disease progression and drug development. Blood plasma and blood cells are important peripheral sources for NDDs’ biomarkers that, although present in lower concentrations than in cerebrospinal fluid, would allow noninvasive diagnostics. To identify new biomarkers for Parkinson’s disease (PD) and amyotrophic lateral sclerosis (ALS), in this work we have evaluated the modifications in the thermodynamic behavior of blood plasma proteome exploring differential scanning calorimetry. The plasma thermodynamics reflected the complexity and heterogeneity of the two pathologies. The unfolding temperature of the most abundant plasma protein albumin and the weighted average center of the calorimetric profile appeared as the two thermodynamic signatures that reflected modifications of the plasma proteome, i.e., strong thermal stabilization of albumin and plasma proteins’ interaction network, related to both pathologies. Based on those two signatures, both PD and ALS patients were stratified in two sets, except several cases with thermodynamic parameters that strongly differed from those of the calorimetric sets. Along with modifications of the plasma thermodynamic behavior, we found altered globulin levels in all PD and ALS patients’ plasma (higher level of α- and β-globulin fractions and lower level of γ-globulin fraction than the respective reference values) employing capillary electrophoresis. The presented results reveal the potential of calorimetry to indirectly identify NDDs’ biomarkers in blood plasma.

## 1. Introduction

The discovery of biomarkers for neurodegenerative diseases (NDDs) is essential for accurate diagnostics, monitoring disease progression, and development of and response to new therapies [1,2,3]. A plethora of investigations have been performed to identify biomarkers—biochemical, imaging, and genetic [4,5,6,7]. In recent years, different biofluids, including cerebrospinal fluid (CSF) and blood, as well as peripheral blood cells were extensively screened for NDDs’ biomarkers [8,9,10,11,12,13,14]. 

Specific proteins, e.g., α-synuclein (α-syn) [15,16,17,18,19] and clusterin [20] in Parkinson´s disease (PD); creatinine, human serum albumin (HSA), and TAR DNA binding protein 43 kDa (TDP-43) in amyotrophic lateral sclerosis (ALS) [21,22,23]; as well as RNAs’ biomarkers [24] have been discovered in biofluids.

The tendency of α-syn to misfold and form aggregates in patients’ brains is critical for PD development [19,25,26,27]. Along with a variety of toxic self-assembled α-syn oligomeric species in PD, the formation of pathological α-syn/Aβ/τ protein assemblies is also related to the disease occurrence [28,29,30,31,32,33]. Several inherited, familial mutations have been found to cause perturbation of the α-syn structure and to correlate with elevated PD risk [34]. Plasma levels of α-syn, Aβ-40, and T-τ are recognized as predictive markers for cognitive decline in PD patients [19,35,36]. Multivariate regression analysis established lower levels of CSF Aβ1-42 and P-τ181 protein in PD patients, while lower levels of CSF T-τ and α-syn in PD patients indicated increased motor severity [29]. Moreover, higher α-syn levels in plasma and sera and CSF neurofilament light chain (NFL) concentrations in PD correlate with disease severity [36,37,38]. 

Metabolomic analysis of human and murine PD plasma discovered increased plasma levels of unconjugated bile acids (cholic acid, deoxycholic acid, and lithocholic acid) and purine-base intermediary metabolites (hypoxanthine) [39], and plasma levels of uric acid were found to be related with the risk and progression of the pathology [40].

Furthermore, NFLs are now increasingly recognized as the most promising candidate biomarker in ALS [41,42]. HSA and creatinine were suggested as independent markers in ALS and are also indicators of disease severity [43]. Albumin in ALS patients was reported to correlate with inflammatory markers, while creatinine with the marker of muscle mass [21]. C-reactive protein and glucose also serve as additional prognostic biomarkers for ALS [43].

A panel technology (multiplex panel of solid-phase proximity ligation assays (SP-PLA)) was applied to analyze CSF samples and identify ALS biomarker candidates among large numbers of proteins [44]. Systematic review and meta-analysis assessed the concentration of commonly reported biomarkers in patients with ALS and ALS subtypes [45]. The presence of peripheral inflammation in ALS and new directions for exploration of biomarkers of inflammation were provided by focusing on peripherally detectable and cellular responses from blood cells [46].

Recently, Michnik et al. [47] reported that differential scanning calorimetry (DSC) can recognize severe stage PD. Here, we further elaborate on the diagnostic potential of this technique for neurodegenerative (PD and ALS) disorders. DSC is a highly sensitive technique for resolving thermally induced conformational transitions of plasma proteins and their binding to other molecules. In addition, the plasma level of HSA and globulins, α-, β-, and γ- fractions, were determined by means of capillary electrophoresis (CE). Although the levels of biomarkers are lower in peripheral blood (plasma and serum) than in CSF, their presence is expected to modify the binding state and conformation of the most abundant plasma proteins and the plasma protein–protein/protein–peptide interaction network that in turn would be reflected in disease-specific plasma calorimetric features. With this purpose, we evaluated a set of thermodynamic parameters of PD and ALS plasma proteome and suggested potential markers that distinguish the studied pathologies from the healthy state.

## 2. Results

### 2.1. DSC of Blood Plasma Derived from Patients with PD and ALS Pathologies

**Figure 1:**
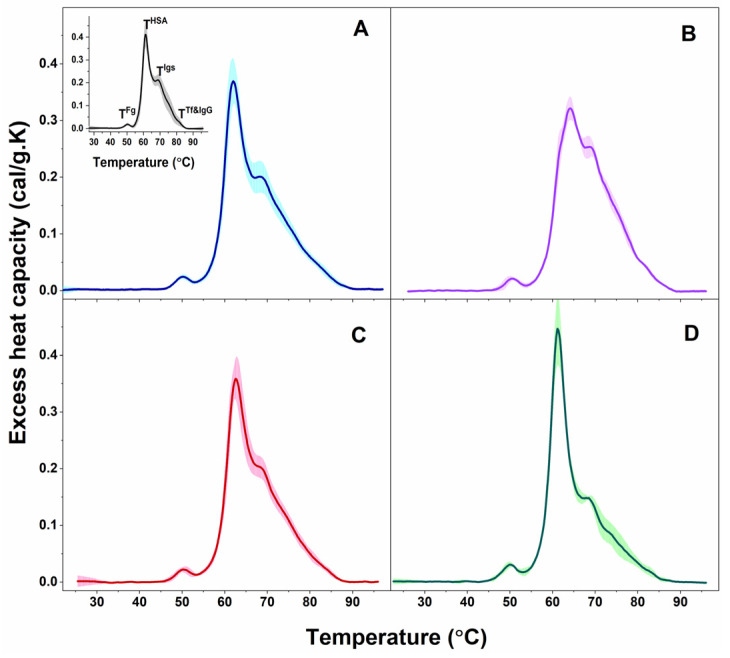
Calorimetric profiles of blood plasma clustered for PD patients in PD1 (**A**) and PD2 (**B**) sets and for ALS patients in ALS1 (**C**) and ALS2 (**D**) sets. Mean scans (solid lines) and SD (shadows). For comparison, the average plasma scan and SD of plasma from healthy individuals (black solid line and grey shadow) are shown in inset of panel A; the temperatures of the thermal transitions assigned to the most abundant plasma protein (T^Fg^, T^HSA^, T^Igs^, and T^Tf&IgG^) are also denoted.

Blood plasma is a complex biofluid composed of many proteins and peptides, among other components, for which conformation and binding states are affected in different pathologies and in turn reflected in the thermodynamic behavior of the plasma proteome. Similar to the data reported previously for healthy individuals [48,49,50], several transitions were resolved in the calorimetric profiles of blood plasma from PD and ALS patients (Figure 1).

The observed transitions were denoted T^Fg^, T^HSA^, T^Igs^, and T^Tf&IgG^, according to their assignment to the most abundant plasma proteins: T^Fg^ to fibrinogen (Fg), T^HSA^ to albumin (HSA), T^Igs^ to immunoglobulins (Igs), and T^Tf&IgG^ to transferrin (Tf) and immunoglobulin G (IgG). Proteins, present in lower concentrations in plasma (including complement proteins, haptoglobin, α-2-macroglobulin, α-1-antitrypsin, α-1-chymotrypsin, and others) can also contribute to these thermal transitions but to a small extent considering the temperatures of their denaturation in isolated state [48,49]; however, their effect is beyond the scope of this work. The thermodynamic behavior of PD and ALS plasma, i.e., the calorimetric profiles and the determined thermodynamic parameters, manifested diversity reflecting the clinical heterogeneity of the two pathologies. Based on the albumin transition temperature, T_m_^HSA^, and the weighted average center or first moment of the thermograms (a measure of the spreading of the calorimetric profile area relative to the temperature), T_FM_, both the PD and ALS cases were clustered in two distinct sets: PD1 (9 patients) and PD2 (5 patients), and ALS1 (7 patients) and ALS2 (3 patients), respectively (mean scans and SD are given in Figure 1A,B for PD and Figure 1C,D for ALS sets).

**Figure 2:**
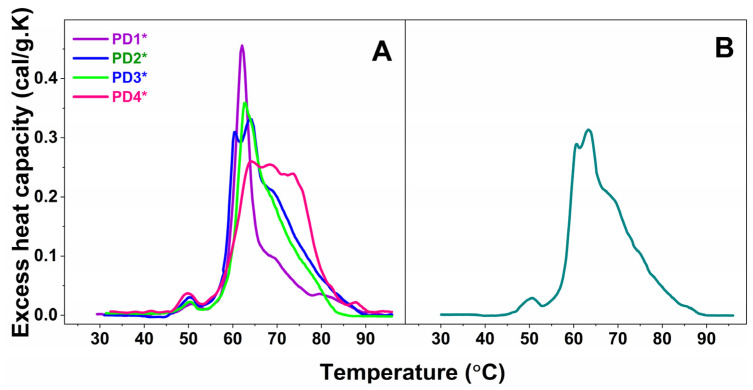
Calorimetric profiles of blood plasma for four not-classified PD cases, denoted PD1*—PD4* (**A**), and one ALS case, denoted ALS* (**B**).

Furthermore, the plasma calorimetric profiles of only four PD cases, denoted PD1*—PD4* and one ALS case, denoted ALS*, shown in Figure 2A and 2B, respectively, differed drastically from those classified in the sets and were not stratified.

The values of the transition temperatures and the corresponding excess heat capacities of the thermal transitions, and the ratio of the heat capacities of albumin and immunoglobulins transitions, determined from analysis of the PD and ALS plasma thermograms, are summarized in Table 1 and compared to those of healthy subjects.

The first thermal transition corresponding to Fg occurred at ca. 50 °C for all PD and ALS cases as found for healthy plasma as well, the c_P_^Fg^ was higher for PD3* and PD4* cases and the ALS2 set than for healthy control, while the other PD and ALS cases had similar values to the healthy ones (Table 1).

**Figure 3:**
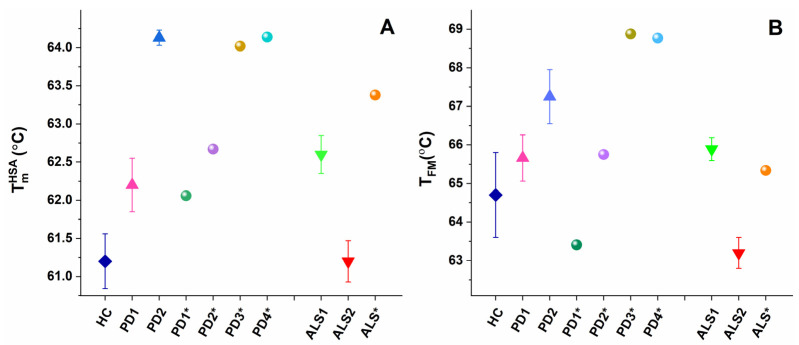
Temperature of albumin unfolding, T_m_^HSA^ (**A**), and the weighted average center of the thermogram, T_FM_ (**B**), for PD and ALS sets (mean and SD) and for non-stratified PD1*–PD4* and ALS* cases.

The main (albumin) transition, centered at ca. 61 °C for healthy plasma, was upshifted by ca. 1 °C for the PD1 set, PD1*, and PD2* and by 3 °C for the PD2 set and PD3* and PD4* cases (Table 1, Figure 3A) suggesting albumin stabilization in PD plasma. Likewise, the albumin was stabilized for the ALS1 set and ALS*, but not for the ALS2 set (Table 1, Figure 3A). Except for the PD1* and ALS2 sets, the heat capacity of albumin transition, c_P_^HSA^, had lower value compared to that of healthy plasma (Table 1).

It is to be noted that in the PD2 set the main transition was located at 64.13 °C, and a shoulder preceding this transition was resolved at ca. 62.03 °C (denoted T_m_^Sh1^ in Table 1, Figure 3A). 

The albumin transition was followed by that of Igs, where the unfolding temperature in PD and ALS plasma did not significantly differ from the healthy one (Table 1). Analysis of the thermograms showed that Igs are most stable in the PD1* case (T_m_^Igs^ transition was shifted to the highest temperature—69.70 °C, and its c_P_^Igs^ was two times lower than the healthy one) and most unstable for PD2* and PD4* cases with T_m_^Igs^ 68.22 °C and 68.34 °C, respectively (Table 1). The Igs transition was followed by a shoulder in PD2* and PD4* thermograms located at ca. 77.51 °C and 71.68 °C, respectively (denoted T_m_^Sh2^ in Table 1).

The Tf&IgG peak was not resolved for all cases, but for eight PDs and two ALSs, it was strongly upshifted for PD4* and ALS* and downshifted for the PD1 set and PD1* case relative to the healthy one (Table 1). The low Tf (215 to 380 mg/dL) and IgG (8 to 18 mg/mL) plasma levels, orders of magnitudes lower than that of HSA (3.4 to 5.4 g/dL), are probably the reason that this transition is hardly resolved in some cases.

Furthermore, the ratio of the excess heat capacities of HSA and Igs transitions, c_P_^HSA^/c_P_^Igs^, varied considerably for PD and ALS cases, being drastically high for the PD1* case and for the ALS2 set, too low for the PD4* case, and in the range from 1.4–1.95 for most cases, due to different tendencies in the changes of c_P_^HSA^ and c_P_^Igs^ values (Table 1). Hence, this ratio cannot be considered for PD and ALS stratification, as previously performed for patients with other diseases [50,51].

The calorimetric enthalpy and weighted average center of the thermograms, two parameters characterizing the thermal stability of the protein–protein interaction network, and the statistical parameters evaluated using the algorithm of Fish et al. [52] are given in Table 2. The calorimetric enthalpy was slightly lower for all ALS cases, as well as for the PD1 set and PD1* and PD2* cases, while slightly higher for PD3* and PD4* cases compared to the enthalpy of the healthy control (Table 2). 

The weighted average center, T_FM_, of the calorimetric profiles had significantly higher value in the range from 65.6–68.77 °C for all PD cases, except for the PD1* case (T_FM_ = 63.41 °C) compared to 64.70 °C for healthy control (Table 2, Figure 2B). This indicates that the plasma protein–protein network is strongly stabilized in PD, while it is significantly destabilized only for the PD1* case. Similarly, T_FM_ reflects stabilization of the ALS1 set and ALS* and destabilization of the ALS2 set plasma proteome (Table 2, Figure 2B). 

It is worth commenting on the individual not-stratified thermograms (PD1*–PD4* in Figure 2A and ALS* in Figure 2B). The four PD* (PD1*–PD4*) thermograms were drastically and differently modified, and hence were the thermodynamic parameters (Table 1 and Table 2). 

The PD1* thermogram exhibited unique features: it was highly cooperative; the Igs transition was shifted to 69.62 °C compared to 68.78 °C for the healthy one; and its heat capacity was drastically reduced to 0.09, i.e., more than two times lower than the healthy c_P_^Igs^ (0.21), resulting in a c_P_^HSA^/c_P_^Igs^ ratio of 5, the highest among all studied PD and ALS cases, and 2.5 times higher than the healthy one (Table 2). PD1*, the only case diagnosed at an early stage, was also characterized with the lowest calorimetric enthalpy (ΔH_cal_ = 3.32 kcal/mol) and the most thermally destabilized proteome (T_FM_ = 63.41 °C) compared to all other NDD cases.

The PD2* case exhibited strongly overlapping albumin and globulin transitions; the globulins’ transition was resolved as a shoulder at 77.51 °C, the high temperature region was featureless, the thermogram was less cooperative than PD1*, and oppositely to PD1* the proteome was thermally stabilized.

Characteristic for the PD3* patient were the shift of the main transition to 64.02 °C and the appearance of a transition at ca. 60.44 °C, both with close heat capacities of 0.31 and 0.33, respectively (Figure 1C, Table 1). The 60.44 °C transition might correspond to the unfolding of another plasma protein, presumably a C3 complement protein and/or haptoglobin, resolved as a consequence of disease-related modification of plasma protein–protein interactions (Figure 2A).

Three peaks with very close c_P_^ex^ overlapped in the PD4* thermogram; they were followed by a shoulder and well-resolved peak in the high temperature region (Figure 2A and Table 1). HSA was significantly stabilized (T_m_^HSA^ = 64.14 °C), and the T_FM_ had the highest value of 68.77 °C indicating the strongest stabilization of the protein interaction network in PD4*. Oppositely to PD1*, the c_P_^HSA^/c_P_^Igs^ ratio of the PD4* case, the only cases at advanced stage, was drastically reduced and had the lowest value of 1.0 compared to all studied PD and ALS cases as well as to the healthy one.

Evaluation of the statistical parameter similarity metric, ρ, showed that the PD and ALS thermograms deviated significantly from the reference healthy set of thermograms (Table 2).

### 2.2. Plasma Globulin Content Determined by Capillary Electrophoresis

In parallel to the DSC measurements, we analyzed blood plasma by CE and determined the levels of plasma albumin and globulin fractions (Table 3).

Capillary electrophoresis data show that the albumin level was within the reference limits for all but two cases (Table 3); minor deviations were found in only one PD case (included in the PD1 set) and one ALS case (included in the ALS2 set). However, PD and ALS patients had strongly modified globulins´ region of the electrophoresis profiles reflecting altered levels of the globulin fractions for all of them (Table 3). In most cases, the levels of α1-, α2-, β1-, and β2-globulin fractions were higher than the upper reference limit; the level of γ–globulin was below the lower limit only in three cases (one case included in PD1 set, one in the ALS1 set, and the PD2* case) and only one case stratified in the PD2 set was much higher than the upper reference value (Table 3). For some patients, more than one or two globulin fractions had altered levels.

There were cases with higher values for α-1-, α-2-, β-1-, and β-2- and lower values of γ-globulin in the PD1 set and cases with higher values of β-1-, β-2-, and γ-globulin in the PD2 set (Table 3). Higher β-2-globulin was characteristic for the PD1* and PD3* cases, 11.40 and 7.05, respectively; for PD4* higher α-2-globulin (13.60) and for PD2* lower γ-globulin (7.51) were characteristic.

Regarding the ALS sets: lower values of β-1- and γ- and higher values of β-2-globulin described the ALS1 set, while higher α-2- and β-2- and lower γ-globulin characterized the ALS2 set. For the ALS* case, the plasma levels of α-1- and β-2-globulin were higher than the reference interval, 5.33 and 7.47, respectively.

## 3. Discussion

For more than a decade, DSC has been employed to analyze blood plasma/sera in healthy and pathological states [47,49,51,53,54,55,56,57,58,59]. DSC has also been successfully applied to study drug-induced effects on red blood cells and blood plasma in animal models [60,61], as well as on skeletal muscle, F and G actin in polyneuropathy [62,63]. The calorimetric profiles of blood plasma/sera are sensitive to the presence of ligands/biomarkers that can affect the conformational state and thermal stability of the most abundant plasma proteins registered by DSC [49,64]. The use of DSC in disease diagnostics has an advantage over other methods because it is fast, non-invasive for the patients, and does not require expensive consumables.

In this work, the thermodynamic behavior of blood plasma from patients with PD and ALS was investigated. The heterogeneity of PD and ALS plasma thermograms apparently reflects the clinical complexity and heterogeneity of the two pathologies (Figure 1 and Figure 2). However, our data demonstrate that PD and ALS share some common thermodynamic features indicating stabilization of HSA (T_m_^HSA^) and the plasma protein–protein interaction network (T_FM_) against the thermal challenge. Therefore, both parameters, T_m_^HSA^ and T_FM_ (Figure 3, Table 2), were used for patients’ stratification in calorimetric sets (Figure 1, Table 1 and Table 2). 

Some PD and ALS cases exhibit the same thermodynamic signatures; PD1 and ALS1 sets, and also the PD3* and ALS* cases, have the same values as the temperatures of the thermal transitions, calorimetric enthalpy, and weighted average center of the thermograms. On the other hand, the PD2 set strongly differs from the ALS2 set; the protein interaction network is stabilized in the former, while it is destabilized in the latter; the albumin stability is not altered in the ALS2 set, and it is strongly stabilized in PD2 set. These data suggest on one hand common modification, and on the other hand specific alterations in the plasma proteome and plasma proteins’ thermal stability in PD and ALS. 

The thermodynamic behavior of PD2 set plasma, i.e., the presence of shoulder and main transition in the range from 62–64 °C, might be attributed to splitting of the albumin transition and strong stabilization of a fraction of the albumin. The shoulder, however, might correspond to the unfolding of another plasma protein, complement 3 or haptoglobin, shown to unfold at ca. 60 °C and 62 °C, respectively, in isolated state [48], and for which its transition most probably overlapped with that of HSA in the healthy thermogram.

Importantly, a lower value of c_P_^HSA^ is determined in PD and ALS cases than that in healthy state, although the albumin content in plasma is not reduced (Table 1 and Table 3), and of ΔH_cal_ of most PD and ALS sets/cases (Table 2) suggesting altered binding states of a fraction of albumin and its conformation, as well as of the protein–protein interaction network. These data are consistent with the reported reduced amplitude of the main (albumin) transition and the enthalpy of serum thermogram for advanced PD state [47].

We found no significant changes in the Igs thermal stability, except in two cases, ALS* and PD1* (with strongly reduced c_P_^Igs^, Table 1). Thus, contrary to HSA, the Igs binding states and conformation for almost all of the studied patients were not modified. 

Along with the altered plasma thermodynamic behavior, we revealed significant changes in the levels of all globulin fractions in plasma from all PD and ALS patients (Table 3) that may have implications for the changed thermodynamics and should be a target of further research.

Therefore, we present clear evidence for strongly altered stability of albumin molecules and the protein–protein interaction network in PD and ALS plasma that reflects the impact of plasma ligands/biomarkers. 

It is to be noted that our recent studies exploring imaging and force–distance curve modes of atomic force microscopy revealed strong and specific changes in the morphometric and mechanical parameters of the peripheral blood cells, platelets, and erythrocytes [12,13] in NDD pathologies. Lower membrane surface roughness, area, and height and significantly higher stiffness; different degrees of activation; distinct pseudopodia; and nanocluster formation were common features of platelets from patients with NDDs; the alterations were less pronounced in PD than in ALS [12]. We proved common modification of the surface nanostructure and of morphometric and nanomechanical features of erythrocytes in the studied NDDs relative to healthy cells, as well as aging-induced transformation that followed different aging pathways for NDDs and normal healthy states [13]. We have also successfully applied the calorimetric approach to differentiate NDDs and healthy erythrocytes, and we found altered conformation of the major cytoplasmic protein hemoglobin and the Band3 transmembrane glycoprotein in NDDs that is more pronounced in aged cells than in fresh ones [14].

Therefore, the altered biophysical features of the peripheral blood and peripheral blood cells might indeed serve as additional promising biomarkers for the studied neurodegenerative pathologies.

## 4. Materials and Methods

### 4.1. Patients

Blood plasma samples derived from 29 patients (18 diagnosed with PD and 11 with ALS) and from 16 healthy volunteers with no neurodegenerative pathologies or other disorders were analyzed. Characteristics of the investigated patients and healthy controls are summarized in Table 4. 

The patients with PD, diagnosed according to MDS-PD clinical criteria [65], had bilateral motor symptoms, and the median Hoehn and Yahr stage was 3 (HY range II–IV) [66], except for one case at early and one case at advanced stage (Table 4). No patients with comorbid dementia were included in the cohort. The mean severity score of the selected ALS patients, based on the El Escorial criteria [67], was 34.5 (mild-to-moderate) according to the Revised ALS Functional Rating Scale (ALSFRS-R) [68]; 7 were with clinically definite and 4 with clinically probable and laboratory supported forms of ALS.

The investigation was approved by the ethics committee of the University multiprofile hospital for active treatment in neurology and psychiatry “St. Naum” (UMHATNP), Sofia, Bulgaria. Written informed consent was obtained from all patients that participated in the investigation. 

### 4.2. Blood Plasma Preparation

Blood samples were collected in Venosafe plastic tubes (Plasma gel) and centrifuged at 900 RCF for 15 min; the supernatant (blood plasma) was removed and diluted 5–8 times in PBS buffer. The total protein concentration of the plasma samples was determined as in [69].

### 4.3. Capillary Protein Electrophoresis

Plasma levels of albumin and immunoglobulin fractions (α1-, α2-, β1-, β2-, and γ-globulins) were determined by capillary electrophoresis (MINICAP, Sebia). Plasma proteins are separated by their size-to-charge ratio in a capillary filled with an electrolyte, and their concentrations are determined [70].

### 4.4. Differential Scanning Calorimetry

The calorimetric profiles (thermograms) of blood plasma derived from patients with PD and ALS and from healthy controls were recorded with a scan rate of 1 °C/min in the range from 30–100 °C using a DASM 4 microcalorimeter. The temperature, T_m_, and excess heat capacity, c_P_^ex^, of the thermal transitions in plasma calorimetric curves were determined. The ratios of the heat capacities of the main transition assigned to HSA and the subsequent transition attributed to immunoglobulins (Igs) were also estimated from the recorded calorimetric curves.

From the plasma calorimetric curves, we also determined the calorimetric enthalpy of denaturation (∆H_cal_ = ∫*c_P_dT*, i.e., the integrated area under the heat capacity curve) and the first moment or weighted average center of the thermogram (*T_FM_*):

(1) $$T_{FM}=\int_{T1}^{T2}Tc_{P}^{ex}(T)dT/\int_{T1}^{T2}c_{P}{}^{ex}(T)dT$$

where *T*1 and *T*2 are the initial and final temperatures of the thermogram, respectively [64]. 

All data were analyzed by Origin software routine.

### 4.5. Statistical Analysis

To correlate NDD plasma thermograms with the healthy (reference) set of thermograms we applied the statistical analysis developed by Fish et al. [52]. Two parameters: distance metric, P, (closeness in space at each temperature point) and correlation coefficient, r, (similarity in shape) were determined and combined to obtain the similarity metric, ρ:

ρ = (P^w^ r^2 − w^)^1/2^ (2)

where w is the weight selected to maximize the differences between distinct curve shapes [52]. Values of ρ close to 1 indicate similar thermograms, while deviation from 1 corresponds to low similarity of the tested and reference thermograms.

## 5. Conclusions

This study presents, for the first time, a direct comparison of the thermodynamic behavior of blood plasma derived from patients diagnosed with PD and ALS that reveals common and specific changes in plasma thermodynamic signatures of the two neurodegenerative disorders. We provide new data indicating strong stabilization of albumin and the complex protein interaction network of blood plasma. We also established strong changes in the plasma level of α1-, α2-, β1-, β2-, and γ–globulin fractions for all studied patients that could have a role in modulation of the plasma thermodynamic behavior in the studied pathologies. The thermodynamic indicators of stabilization of HSA (T_m_^HSA^) and the plasma protein–protein interaction network (T_FM_) and the amplitude of HSA thermal transition are identified as possible new markers for the two studied pathologies, which, however, need further validation.

## Figures and Tables

**Table 1:** Thermodynamic parameters (transition temperature, T_m_ (°C) and amplitude, c_P_^ex^ (cal/g.K) of the successive thermal transitions (T_m_^Fg^, T_m_^Sh1^, T_m_^HSA^, T_m_^Igs^, T_m_^Sh2^, T_m_^Tf&IgG^) and the ratios of the excess heat capacities of T_m_^HSA^ and T_m_^Igs^ transitions (c_P_^HSA^/c_P_^Igs^) for healthy, PD, and ALS plasma. The number of cases included in each calorimetric set are given in parenthesis.

Sets	Calorimetric Parameters						
	T_m_^Fg^ (c_P_^Fg^)	T_m_^Sh1^ (c_P_^Sh1^)	T_m_^HSA^ (c_P_^HSA^)	T_m_^Igs^ (c_P_^Igs^)	T_m_^Sh2^ (c_P_^Sh2^)	T_m_^Tf&IgG^ (c_P_^Tf&IgG^)	c_P_^HSA^/ c_P_^Igs^
Healthy (16)	50.50 ± 0.018 (0.017 ± 0.003)		61.20 ± 0.36 (0.41 ± 0.05)	68.78 ± 0.26 (0.21 ± 0.02)		81.45 ± 0.4 (0.03 ± 0.01)	1.95 ± 0.51
PD1 (9)	50.4 ± 0.3 (0.022 ± 0.02)	-	62.2 ± 0.35 (0.36 ± 0.07)	68.77 ± 0.2 (0.19 ± 0.014)	-	80.56 ^Sh^ (0.03)	1.94 ± 0.4
PD2 (5)	50.22 ± 0.4 (0.02 ± 0.004)	62.03 ± 0.3 (0.26 ± 0.02)	64.13 ± 0.1 (0.32 ± 0.02)	68.77 ± 0.7 (0.25 ± 0.02)			1.4 ± 0.1
PD1*	50.70 (0.018)	-	62.06 (0.45)	69.62 (0.09)	-	79.76 (0.018)	5.0
PD2*	50.35 (0.023)		62.67 (0.36)	68.22 ^Sh^ (0.20)	77.51 ^Sh^ (0.073)	-	1.8
PD3*	50.32 (0.03)	60.44 (0.31)	64.02 (0.33)	68.92 ^Sh^ (0.21)	-		1.6
PD4*	49.84 (0.03)		64.14 (0.25)	68.34 (0.24)	73.54 (0.023)	87.84 (0.015)	1.0
ALS1 (7)	50.37 ± 0.4 (0.02 ± 0.003)		62.60 ± 0.6 (0.36 ± 0.004)	68.8 ± 0.5 (0.20 ±0.02)			1.8 ± 0.3
ALS2 (3)	50.06 ± 0.5 (0.03 ± 0.003)		61.20 ± 0.3 (0.44 ± 0.06)	68.54 ± 0.3 (0.15 ± 0.01)	73.86 ± 0.4 (0.08 ± 0.02)		2.9 ± 0.2
ALS*	50.67 (0.02)	60.61 (0.28)	63.38 (0.31)	69.03 (0.19)		86.18 ^Sh^ (0.011)	1.6

**Table 2:** Weighted average center of the thermograms (T_FM_) and calorimetric enthalpy (ΔH_cal_) of healthy, PD, and ALS calorimetric profiles (average values and SD for PD and ALS sets) and statistical parameters (spatial distance metric (P); Pearson‘s correlation coefficient (r) and similarity metric (ρ)) for PD sets, PD1*–PD4* cases (not included in the PD sets), ALS sets, and ALS* case (not included in ALS sets) relative to the healthy ones.

Sets	Parameter				
	T_FM_ (°C)	ΔH_cal_ (kcal/g)	P	r	ρ
Healthy	64.70 ± 1.1	4.60 ± 0.4			
PD1	65.66 ± 0.6	4.43 ± 0.3	0.90	0.92	0.91
PD2	67.25 ± 0.7	4.60 ± 0.2	0.82	0.64	0.77
PD1*	63.41	3.32	0.76	0.88	0.78
PD2*	65.75	4.02	0.86	0.67	079
PD3*	65.88	4.86	0.88	0.81	0.83
PD4*	68.77	4.81	0.72	0.34	0.60
ALS1	65.89 ± 0.3	4.25 ± 0.4	0.82	0.98	0.86
ALS2	63.20 ± 0.4	4.00 ± 0.2	0.81	0.68	0.77
ALS*	65.34	4.38	0.83	0.90	0.85

**Table 3:** Albumin and globulin concentrations (mean values and SD, and individual values) are shown in percentage of the total plasma protein for PD and ALS patients. The level of proteins out of the reference ranges is given in bold. The numbers in superscript denote the number of cases for the given set with certain protein level(s) out of the reference limits. The number of patients classified in sets is given in parentheses.

Sets	Albumin (%)	Globulins (%)				
		α-1	α-2	β-1	β-2	γ
	Reference Ranges					
	55.8–66.1	2.9–4.9	7.1–11.8	4.7–7.2	3.2–6.5	11.1–18.8
PD1 (9)	57.74 ± 1.61 **48.14** ^1^	5.94 ± 0.37 **5.21–6.42** ^3^	13.15 ± 0.8 **12.35–13.95** ^2^	8.68 ± 0.26 **8.41–8.94** ^2^	8.30 ± 0.72 **6.60–10.84** ^6^	12.81 ± 0.90 **7.78** ^1^
PD2 (5)	57.28 ± 0.82	3.76 ± 0.08	8.94 ± 0.23	6.55 ± 1.30 **9.09** ^1^	5.23 ± 1.35 **7.88** ^1^	18.22 ± 1.98 **21.48** ^1^
PD1*	56.80	4.00	10.00	6.50	**11.40**	11.30
PD2*	64.80	4.71	10.39	6.56	6.04	**7.51**
PD3*	57.68	4.42	11.49	6.14	**7.05**	13.23
PD4*	58.35	4.42	**13.60**	**7.26**	**6.24**	**10.13**
ALS1 (7)	61.41 ± 0.93	4.29 ± 0.14	10.39 ± 0.37 **11.82** ^1^	5.16 ± 0.79 **2.10** ^1^	5.45 ± 0.22 **7.11** ^1^	12.80 ± 1.28 **8.87** ^1^
ALS2 (3)	61.68 ± 2.73 **69.25** ^1^	3.96 ± 0.37	10.73 ± 1.01 **13.02** ^1^	6.12 ± 0.46	6.53 ± 0.83 **8.63** ^1^	12.27 ± 1.15 **8.83–10.91** ^2^
ALS*	59.76	**5.33**	9.01	7.18	**7.47**	11.25

**Table 4:** Characteristics of patients and healthy controls.

	Healthy	PD	ALS
Number of patients	16	18	11
Gender, M/F	7/9	8/10	6/5
Age (years) mean (SD)	63 ± 5.8	66.9 ± 2.1	60.4 ± 3.9
Range (years)	51–73	59–78	42–78
Severity score	-	1 patient at early stage (denoted PD1*) 16 patients with median Hoehn and Yahr stage 1 patient at advanced stage (denoted PD4*)	mild-to-moderate

## Data Availability

Data supporting this study are available upon reasonable request.
